# Chromosome replication as a measure of bacterial growth rate during *Escherichia coli* infection in the mouse peritonitis model

**DOI:** 10.1038/s41598-018-33264-7

**Published:** 2018-10-08

**Authors:** Maria Schei Haugan, Godefroid Charbon, Niels Frimodt-Møller, Anders Løbner-Olesen

**Affiliations:** 1 Department of Biology, University of Copenhagen, Copenhagen, Denmark; 2 Department of Clinical Microbiology, Rigshospitalet, Copenhagen, Denmark

## Abstract

The efficacy of most antibiotics is dependent on active bacterial growth, yet little is known about the growth dynamics during infection. Therefore, means to measure in-host bacterial growth rate is of importance. Here, we use chromosome replication as readout for *in situ* bacterial growth rate during infection; obtained from a single biological specimen. We have applied two independent methods: quantitative PCR (qPCR) and fluorescence microscopy, to quantify the level of chromosome replication present during *Escherichia coli* propagation in the mouse peritonitis model. We find that the methods complement each other and allow for quantification of growth rate, both on a population average and on a single-cell level. We demonstrate the presence of heterogeneous growth rates within bacterial populations propagating during infection. Also, no growth cessation was observed during the apparent stationary phase *in vivo*, and, by comparison of growth dynamics at different anatomical sites, we demonstrate that *E. coli* is unlikely to grow independently intravascularly. These findings provide novel insight into bacterial growth during host infection, and underscore the importance of pinpointing the primary site of infection in septicaemia of unknown origin and ensuring antibiotic availability at this site.

## Introduction

The interplay between bacterial growth and chromosome replication has been studied extensively under laboratory conditions for decades^1–6^. Until recent years, the detection of chromosome replication status in bacteria propagating in their natural host (*in vivo)* had received scant attention. Bacterial growth *in vivo* has generally been assessed by viable bacterial counts (colony forming units (CFU)/ml)) plotted as a function of time. In a batch culture with a finite amount of nutrients, this curve is divided into well-defined growth phases^7^. However, bacterial growth dynamics *in vivo* are complex; growth rate is not solely a function of nutrient availability, but also of host immune-mediated clearance^8–11^. Applying bacterial count measurements to deduce an absolute bacterial growth rate *in vivo* is therefore not adequate, as this merely reflects the net change in bacterial population size. Neither does it reflect the level of growth rate heterogeneity within the population. To circumvent some of these limitations, recently published studies have explored the use of chromosome replication status as readout for bacterial growth rate in environmental and human microbiomes and found good correlation between the two^12–14^. However, this was achieved by whole genome sequencing, a method that besides being expensive and time-consuming, only provides a population average readout and fails to provide information on any possible differential growth rates within the population.

*Escherichia coli*, like many other bacteria, possesses one circular chromosome. DNA replication is initiated from a single origin of replication (*oriC)* and carried out bidirectionally, terminating in the opposite replication terminus region (*terC)*^1,2,15,16^. Coordination between chromosome replication and cell division ensures that the two identical daughter cells accommodate at least one fully replicated chromosome^17,18^. Initiation of replication takes place at a fixed cell mass per origin: the initiation mass^3,19–22^. Under favourable growth conditions multiple rounds of synchronously initiated replications per division cycle can occur^4,23^. This allows for faster growth rates, with daughter cells carrying chromosomes with overlapping rounds of replication, i.e. more than one number of *oriC* copies (2^n^, n = 1, 2, 3), at birth. Due to the consistency of the initiation mass, rapidly growing cells harbouring overlapping rounds of replication have larger cell mass proportional to the number of *oriC* copies. The growth rate of the population is reflected by the copy number ratio of *oriC* to *terC* (*ori:ter*), with an *ori:ter* of 1 representing non-replicating cells carrying a complete set of chromosome(s) and an *ori:ter* > 1 representing cells with ongoing chromosome replication (growing cells)^1,24^. Theoretically, this correlation can be expressed as *ori:ter* = 2 ^C/τ^ (1), where C is the chromosome replication time (C-period) and τ is the mass doubling time, and thus τ = C/log_2_(*ori:ter*) (2)^24^. However, such an unambiguous measure can only be inferred from a population of bacterial cells propagating at balanced growth rates, and only at τ < approximately 60 minutes, where C has been shown to be constant^25^.

Here, we applied quantitative PCR (qPCR), an inexpensive and easily accessible method, to provide population average measurements of bacterial growth rates during infection *in vivo*, combined with chromosomal fluorescent marker frequency analysis for quantification of bacterial growth at a single-cell level. The goal was to determine variation in *in situ* bacterial growth rates at various anatomical sites, which would add to our current understanding of growth dynamics during infection. We hypothesized that growth dynamics within and outside the primary site of infection would be different. Furthermore, the detection of an *in situ* bacterial growth rate obtained from one single specimen could form the basis for refining future antibacterial strategies, knowing that the efficacy of most antibiotics in clinical use is related to active bacterial growth^26,27^.

## Results

**Figure 1:**
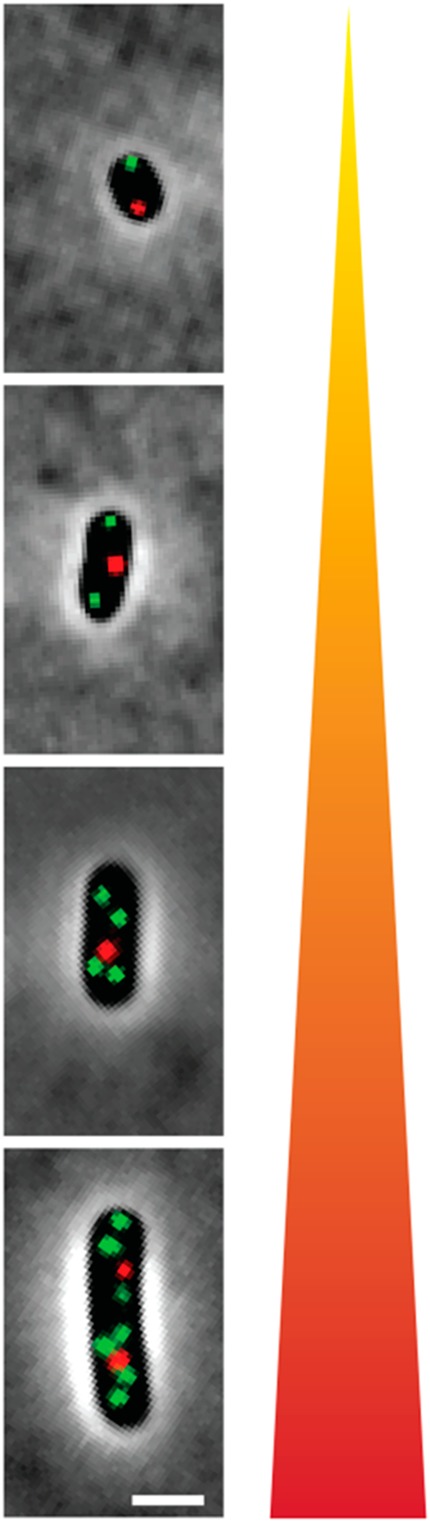
Visualisation of intracellular *oriC* foci (green) and *terC* foci (red) by fluorescence microscopy in ALO 4783 (shown in phase contrast) growing at various growth rates; from rapid (bottom) to slow (top) growth. Scale bar is 2 µm.

Two methods for detection of *ori:ter* were tested *in vivo* in an experimental murine infection model; qPCR, where the *ori:ter*_qPCR_ was given as a population mean, and fluorescence microscopy, where the *ori:ter*_mic_ was deduced from direct analysis of single fluorescently labelled live bacterial cells (exemplified in Fig. 1, where *oriC* foci are displayed in green (GFP labelling) and *terC* foci are displayed in red (mCherry labelling)). In *ori:ter*_qPCR_ the ‘*oriC*’ copy number was inferred from a site located immediately to the left of *oriC*, and the ‘*terC*’ copy number was inferred from a site located in close proximity to *terC*^28^. We applied these methods, in combination with bacterial counts, to reveal fundamental bacterial growth dynamics at various anatomical sites during infection. Furthermore, the correlation between cell length (µm) (measured by microscopy and used as a proxy for cell mass) and *oriC*/cell was measured to investigate initiation mass consistency during infection *in vivo*. As *proof-of-concept*, the same methods were tested and validated *in vitro*, using the same model infective organism; *Escherichia coli* ATCC® 25922™.

### Bacterial growth *in vitro*

Bacteria grown *in vitro* in Lysogeny Broth (LB) batch cultures were used to validate the methods applied in the *in vivo* experiments. Here, stationary phase bacterial cells were diluted in fresh media and allowed to grow with repeated sample collections at time points 2, 4, 6, 8 and 10 hours of incubation. As a complete cessation of growth was observed at 10 hours of incubation, the *in vitro* experiments were not extended beyond this time point. We tested the wild-type strain (ATCC 25922) alongside a genetically modified derivative of the strain, with chromosomally incorporated fluorescent *oriC* and *terC* labels (ALO 4783). This was done to ensure that transgene insertion would not affect growth dynamics of the derivative strain, which was applied in the *in vivo* infection model.

**Figure 2:**
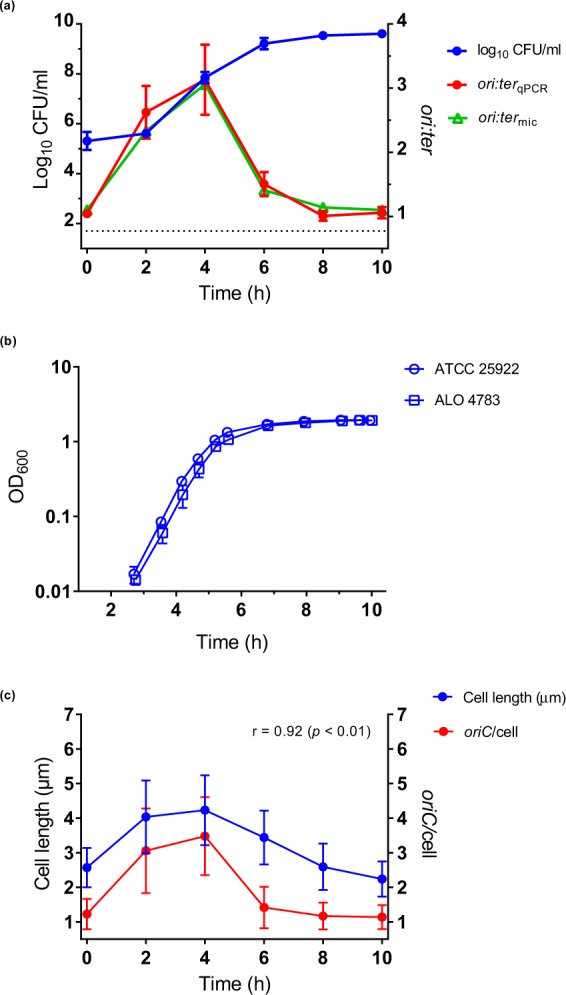
Bacterial growth in LB batch cultures (*in vitro)*. (**a**) Bacterial growth measured as bacterial counts (log_10_ CFU/ml), *ori:ter*_qPCR_ and *ori:ter*_mic_. ATCC 25922 and ALO 4783 were tested in parallel and showed similar growth; hence, results from both strains are pooled in the data sets. Bland-Altman agreement between *ori:ter*_qPCR_ and *ori:ter*_mic_ was good (Supplementary Fig. S1). 6 h, n = 12; all other time points, n = 6. (**b**) Comparisons of ATCC 25922 and ALO 4783 grown in LB batch cultures (*in vitro)* revealed no growth retardation due to transgene insertions. Growth measured as OD_600_. Both strains exerted doubling times of 25 minutes during exponential growth. (**c**) Bacterial cell length (µm) (used as proxy for cell mass) correlate with *oriC*/cell. Data represent pooled microscopically detected cells (ALO 4783). n = 500 at all time points, with the exception of 2 h, where n = 147. Data are presented as mean ± SD. Dotted line represents the limit of detection for bacterial count measurements. Time (h) represents hours of incubation; inoculum is presented as 0 h. r: Pearson’s correlation coefficient.

Growth curves from *in vitro* studies are presented as bacterial counts as a function of time in Fig. 2a, and as OD_600_ values as a function of time in Fig. 2b, respectively. Exponential growth was observed between the initial sampling time point and approximately 6 hours of incubation, after which growth progressively ceased towards stationary phase. The mass doubling time (τ) inferred from mean OD_600_ measurements during exponential growth was 25 minutes. No growth reduction due to transgene insertions in ALO 4783 was observed (Fig. 2b).

**Figure 3:**
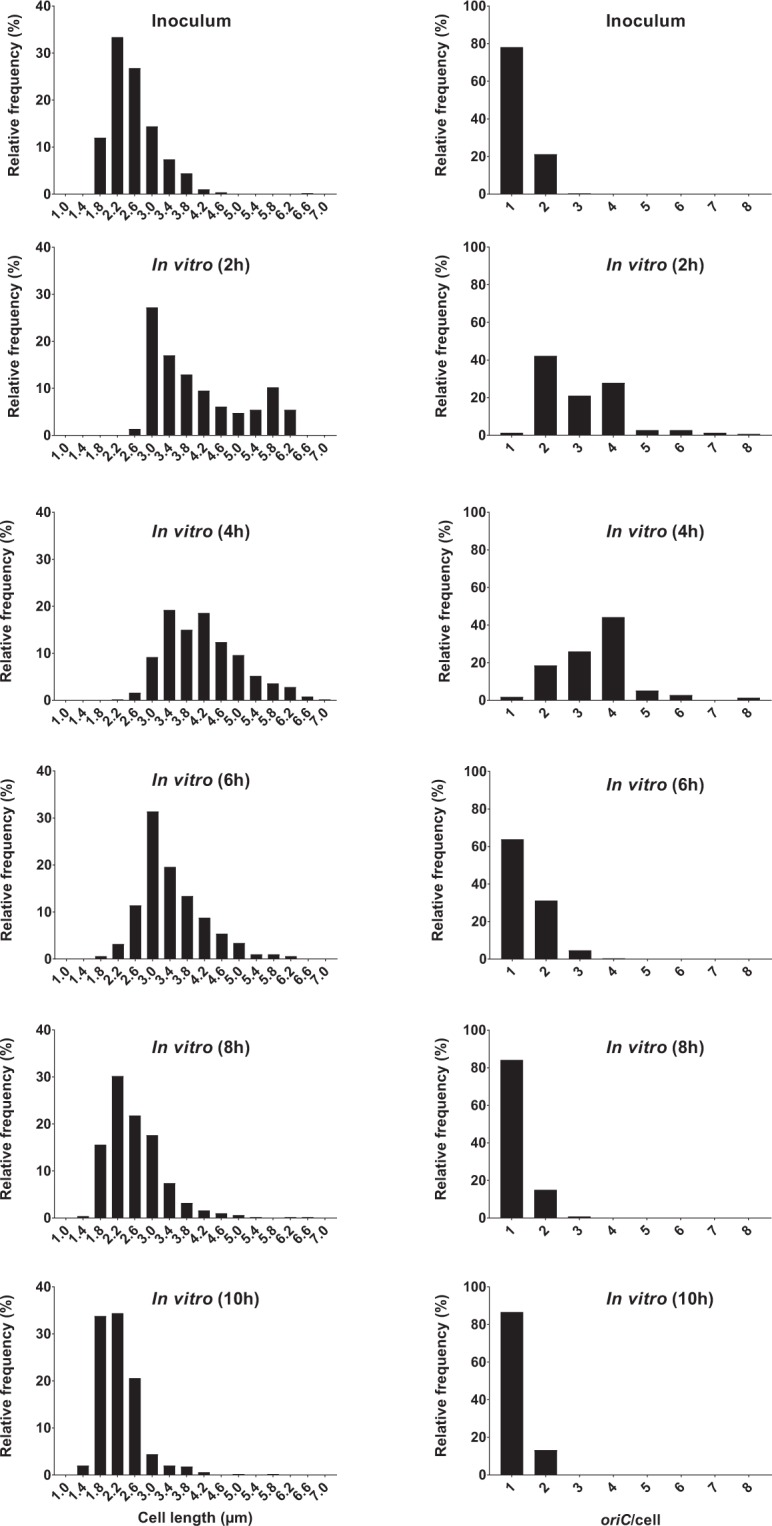
Relative frequency distributions (%) of pooled microscopically detected bacterial cells (ALO 4783) from growth in LB batch cultures (*in vitro)*; cell length (µm) (left panel) and *oriC*/cell (right panel). n = 500 at each time point, with the exception of 2 h, where n = 147. Time (h) represents hours of incubation. For *oriC*/cell histograms, note that data are presented as detected by automated foci quantification, which is subject to the risk of underestimation due to co-localising *oriC*s^48^. Hence, it is likely that 3 detected foci truly represent 4 foci and that >4 detected foci truly represent 8 foci (see Methods).

The two methods for detection of chromosome replication status (*ori:ter*_qPCR_ and *ori:ter*_mic_) were in good agreement (Supplementary Fig. S1). *ori:ter*_qPCR_ and *ori:ter*_mic_ followed growth rate (Fig. 2a, Supplementary Table S1). We observed a rapid increase in *ori:ter* during the first 4 hours, followed by a gradual decline as the cells stopped growing by 8 hours; remaining unchanged at 10 hours of incubation. In accordance with these findings, single-cell fluorescence microscopy analysis of *oriC*/cell and cell size (µm) confirmed a shift from a homogenous bacterial population dominated by non-growing, small cells at 0 hours (i.e. the inoculum; *oriC*/cell ~1), to a heterogeneous population dominated by bacterial cells with ongoing chromosome replications at various levels (*oriC*/cell ≥ 2) and various cell sizes at 2 hours of incubation (Fig. 3). Starting from 4 hours of incubation, a tendency towards more growth homogeneity was observed. At this time point, an *oriC*/cell of 4 was observed in approximately 80 percent of the population, reflecting close to balanced growth (Fig. 3). Here, criteria for inferring mass doubling time (τ) from *ori:ter* were met, and τ was computed from equation (2) as (median (IQR)) 23.4 (21.5–29.9) minutes (assuming a constant C period of 40 minutes)^25^. Almost no overlapping rounds of replication were observed starting from 6 hours of incubation (*oriC*/cell ≤ 2). At 8 and 10 hours of incubation homogenous populations of small bacterial cells harbouring predominantly one copy of *oriC*/cell were observed (Fig. 3). The correlation between *oriC*/cell and cell size (µm) was very strong (*r* = 0.92, *p* < 0. 01) (Fig. 2c).

A slow-down of growth was initiated between 4 and 6 hours of incubation, during which period we observed a decrease in both *oriC*/cell and *ori:ter*, manifesting cessation of chromosome replication (Fig. 2a,c). As expected^1^, cell division proceeded for some time longer, as demonstrated both by the bacterial count increase and cell size decrease (Fig. 2a,c). Eventually, no new initiations of chromosome replication took place during stationary phase (8–10 hours of incubation). Inferring τ from *ori:ter* for time points later than 4 hours of incubation would be deceptive, given that the growth was too slow (i.e. the criteria of τ < 60 minutes was not met).

We can conclude that, when these bacterial cells were growing fast the *ori:ter* and *oriC*/cell ratios were high and cell sizes were large, and when cells were growing slowly the *ori:ter* and *oriC*/cell ratios were low and cell sizes were small. Our findings are consistent with the existence of a fixed initiation mass at all *in vitro* growth rates, exemplified by the good correlation between *oriC*/cell and cell length (Fig. 2c). These data are consistent with previous studies that have shown correlation between *ori:ter* and initiation mass in *E. coli* K-12 strain MG1655 under various growth conditions *in vitro*^29^. At mid-exponential phase (4 hours of incubation; Fig. 2a) a mass doubling time (τ) could be inferred from *ori:ter* and was similar to that inferred from OD_600_ measurements; approximately 23 and 25 minutes, respectively.

### Bacterial growth *in vivo*: mouse peritonitis model

Bacterial growth dynamics at separate anatomical sites during widespread bacterial infection were evaluated in the model of lethal peritonitis in mice, aiming both to validate the *ori:ter* method for use *in vivo*, and to determine any potential growth rate differences within and outside the primary site of infection.

An intraperitoneal (i.p.) dose of 1 × 10^6^ CFU/ml stationary phase bacteria (ALO 4783) resulted in the establishment of septicaemia in a total of 51/51 animals within 2 hours of infection. Samples (peritoneal lavage fluid (PLF), blood, spleen and kidneys) were collected in biological triplicates at time points 2, 4, 6, 8 and 10 hours of infection. After 10 hours of infection animals demonstrated signs of distress to such an extent that criteria for euthanasia were met. Thus, this constituted the final possible sample collection time point in this model. Bacterial DNA and live bacterial cells were successfully isolated from PLF and blood for qPCR and microscopy analysis, respectively. Unfortunately, we were unable to adequately isolate bacterial DNA or live bacterial cells for the above mentioned analyses from spleen and kidneys. Consequently, the PLF and blood bacterial populations constituted representative populations of bacteria growing within and outside of the primary site of infection, respectively. It has previously been hypothesised that bloodstream bacterial populations represent a passive ‘spill-over’ of bacteria from the primary site of infection, rather than independently growing populations^30^. Using chromosome replication as readout for *in situ* bacterial growth rate, we aimed at improving insight into whether these populations truly differ or not.

**Figure 4:**
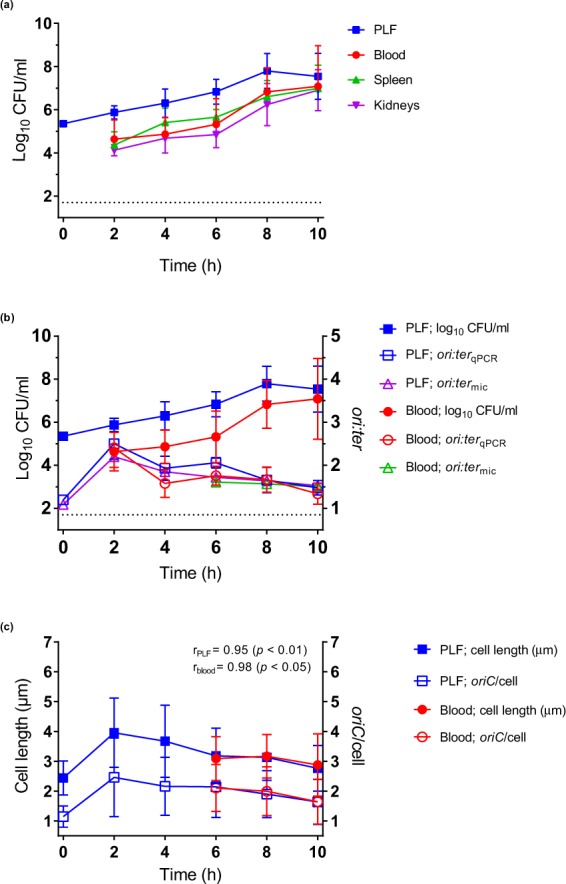
Bacterial growth (ALO 4783) in the mouse peritonitis model (*in vivo*). (**a**) Bacterial growth measured as bacterial counts (log_10_ CFU/ml) in peritoneal lavage fluid (PLF), blood, spleen and kidneys: 2 h, n = 15; 4 h, n = 9; 6 h, n = 6; 8 h, n = 12; 10 h, n = 9. Slopes (β) from linear regression analysis (0–8 h; blood, spleen and kidneys versus PLF) were not significantly different (*p* > 0.05), yet elevations (blood, spleen and kidneys versus PLF) were all significantly different (*p* < 0.0001) (**b**) Bacterial growth measured as bacterial counts, *ori:ter*_qPCR_ and *ori:ter*_mic_ in PLF and blood. PLF bacterial count and *ori:ter*_qPCR_: 2 h, n = 15, 4 h, n = 9; 6 h, n = 6; 8 h, n = 12; 10 h, n = 9. Blood bacterial count: 2 h, n = 15, 4 h, n = 9; 6 h, n = 6; 8 h, n = 12; 10 h, n = 9. Blood *ori:ter*_qPCR_: 2 h, n = 12; 4 h, n = 8; 6 h, n = 5; 8 h, n = 12; 10 h, n = 9. *ori:ter*_mic_ from 2 and 4 h (blood) are not presented due to insufficient number of microscopically detected cells (Supplementary Table S1). Bland-Altman agreement between *ori:ter*_qPCR_ and *ori:ter*_mic_ was good (Supplementary Fig. S1). (**c**) Bacterial cell length (µm) (proxy for cell mass) correlate with *oriC*/cell, both in PLF and blood. Data represent pooled microscopically detected cells. Inoculum (0 h), n = 500. PLF: 2 h, n = 133; 4 h, n = 55; 6 h, n = 132; 8 h & 10 h, n = 500. Blood: 6 h, n = 164; 8 h, n = 157; 10 h, n = 500; 2 h and 4 h time points are not presented due to insufficient number of cells. Data are presented as mean ± SD. Dotted line represents the limit of detection for bacterial counts. Time (h) represents hours of infection; inoculum is presented as 0 h and extrapolated to PLF data. r: Pearson’s correlation coefficient.

Pooled bacterial count data from repeated *in vivo* experiments demonstrated that the net size of the bacterial populations found outside the primary site of infection (i.e. bloodstream, spleen or kidneys) increased in parallel with the bacterial population at the primary site of infection (i.e. peritoneum) (Fig. 4a). For all collected specimens (PLF, blood, spleen and kidneys) a pronounced increase in bacterial count was observed from 0 to 8 hours of infection, indicating bacterial growth. There was no apparent lag phase, however, this cannot be ruled out since no measurements were made prior to 2 hours of infection. Linear regression analysis derived slopes (β) for growth in blood, spleen and kidneys revealed no significant difference from growth in PLF (*p* > 0.05), whereas the blood, spleen and kidney bacterial counts differed significantly from the PLF bacterial count (*p* < 0.0001); meaning that bacterial population sizes outside the PLF were smaller, yet increasing in parallel with the PLF bacterial population (Fig. 4a). Also, attention should be drawn to the fact that the bacterial count found at the primary site of infection was under-estimated. The PLF bacterial count was in reality substantially higher than reported, taken into consideration the dilution factor from the required addition of lavage fluid during harvesting (see Methods). From 8 to 10 hours of infection the net bacterial count was stagnant (Fig. 4a).

The maximum *ori:ter*_qPCR_ (mean (SD)) measured *in vivo* (2.50 (0.36) in PLF) was lower than the maximum *ori:ter*_qPCR_ measured *in vitro* (3.13 (0.55)), accounting for an overall slower growth *in vivo* (Figs 4b and 2a, Supplementary Table S1). Nevertheless, *ori:ter* followed overall growth rate *in vivo* (Fig. 4b, Supplementary Table S1). In the PLF *ori:ter*_qPCR_ increased rapidly during the first 2 hours, followed by a slight decline towards unchanged levels at 4 and 6 hours of infection. After 6 hours of infection a gradual decline towards an *ori:ter*_qPCR_ approaching 1 was observed (Fig. 4b, Supplementary Table S1). In the blood, *ori:ter*_qPCR_ did not change between 4 and 8 hours. However, no significant difference (*p* > 0.05) in *ori:ter*_qPCR_ were found between PLF and blood, when compared both overall by linear regression analysis and time-point by time-point (2 to 10 hours of infection) (Fig. 4b).

**Figure 5:**
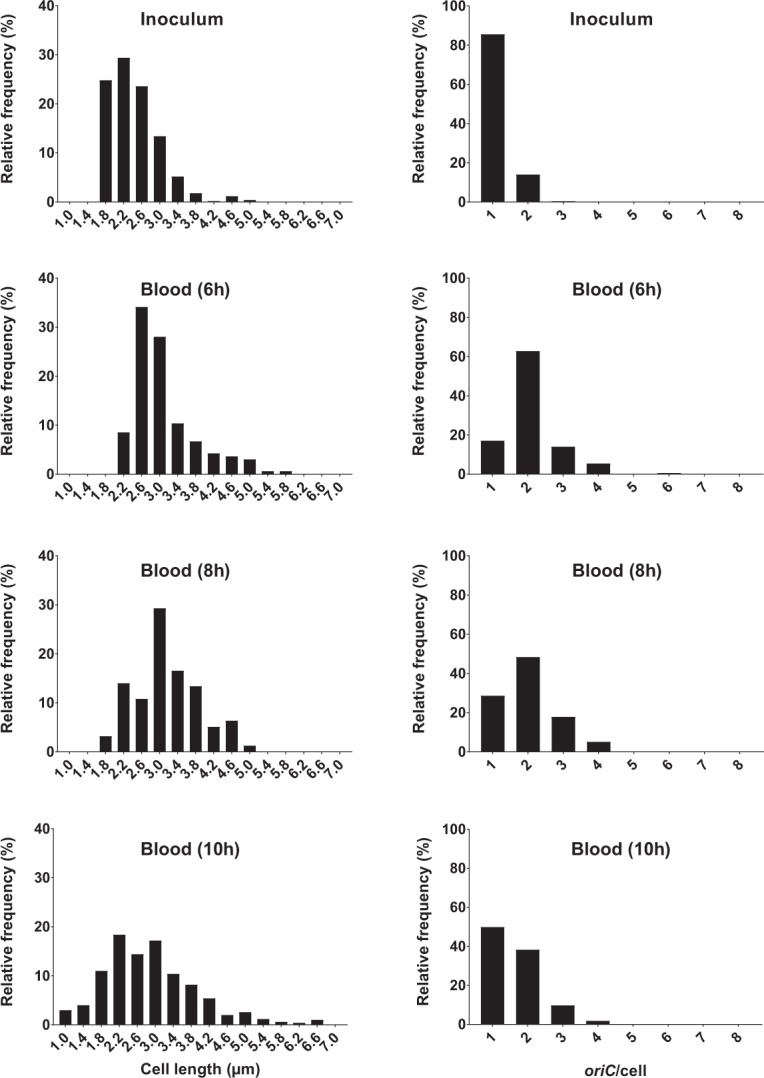
Relative frequency distributions (%) of pooled microscopically detected bacterial cells (ALO 4783) isolated from blood in the mouse peritonitis model; cell length (µm) (left panel) and *oriC*/cell (right panel). Inoculum, n = 500; 6 h, n = 164; 8 h; n = 157; 10 h, n = 500. Data from 2 and 4 h are not presented, due to insufficient number of microscopically detected cells (Supplementary Table S1). Time (h) represents hours of infection.

**Figure 6:**
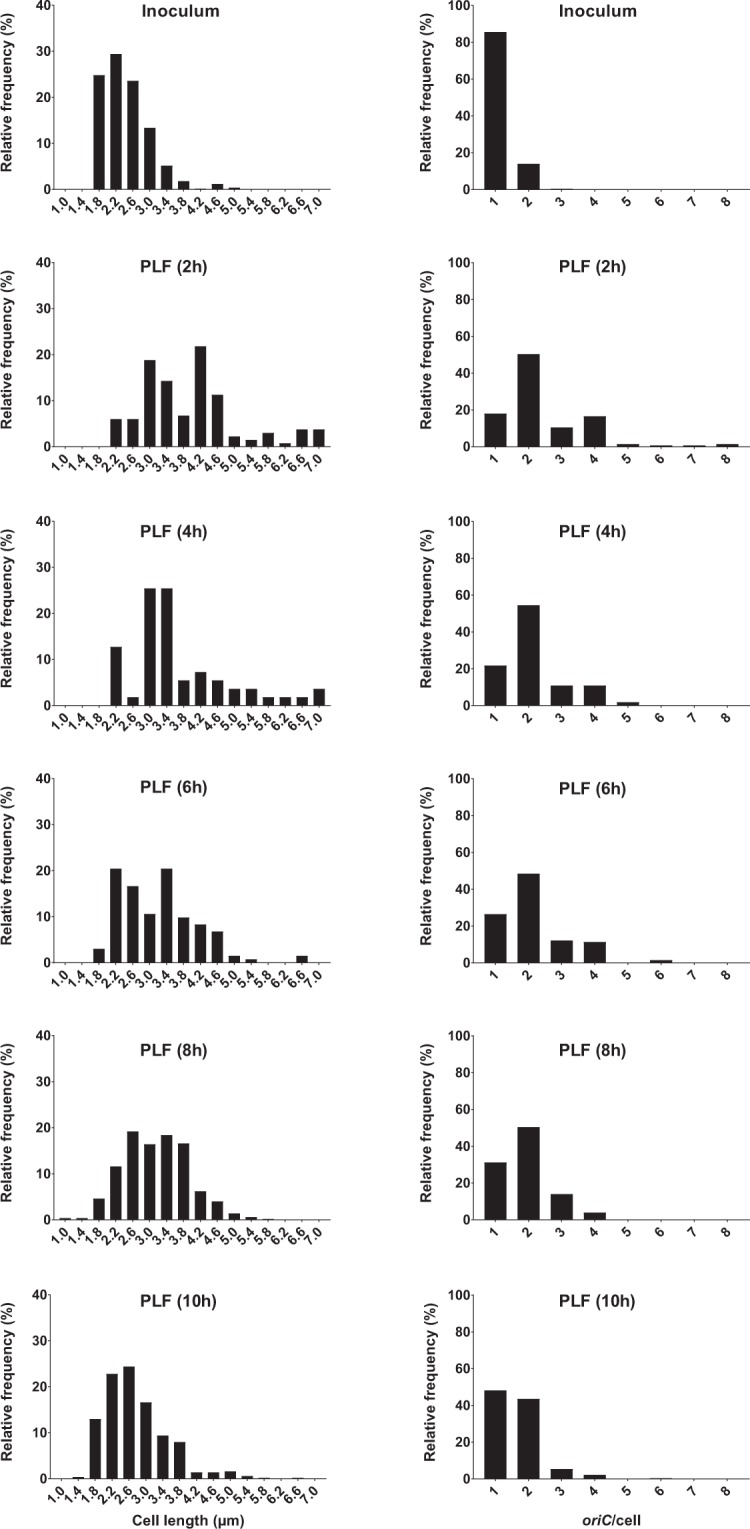
Relative frequency distributions (%) of pooled microscopically detected bacterial cells (ALO 4783) isolated from peritoneal lavage fluid (PLF) in the mouse peritonitis model; cell length (µm) (left panel) and *oriC*/cell (right panel). Inoculum, n = 500. PLF: 2 h, n = 133; 4 h, n = 55; 6 h, n = 132; 8 h, n = 500; 10 h, n = 500. We emphasize that data representing 4 hours of infection are subject to uncertainty, due to low number of detected cells (n < 100). Time (h) represents hours of infection.

There was no significant difference (*p* > 0.05) in *oriC*/cell or cell size (µm) between PLF and blood bacterial populations, compared at 6, 8 and 10 hours of infection (Fig. 4c). At 2 and 4 hours of infection, the total number of microscopically detected live bacterial cells from the blood was too low. Hence, these data are not included in statistical analyses or illustrations (Supplementary Table S1, Figs 4c and 5). *oriC*/cell and cell size (µm) data demonstrated a shift from a nearly homogenous bacterial population dominated by non-growing, small cells at 0 hours (i.e. the inoculum; *oriC*/cell ~1), to heterogeneous populations dominated by bacterial cells with ongoing chromosome replications at various levels (*oriC*/cell ≥ 2) and various cell sizes at 2 (PLF only), 4 (PLF only), 6 hours of infection (PLF and blood) (Figs 5 and 6). A minor decline in both *oriC*/cell and cell size was seen at 8 hours, and was even more pronounced at 10 hours of infection. However, unlike during stationary phase *in vitro*, we did not observe a complete cessation of chromosome replication during the length of infection. At 10 hours of infection (where minimal *oriC*/cell and cell size were observed) PLF and blood *oriC*/cell were significantly different from inoculum *oriC*/cell (*p* < 0.0001) (Figs 5 and 6). This indicated that fractions of the population were still growing, albeit slowly, at the terminal stage of infection. It has previously been reported, however, that bacteria may cease growth with 2 *oriC*/cell present^31^. Yet, under such conditions, *ori:ter* would go toward 1 (i.e. 2 fully replicated chromosomes), which we do not observe here.

The correlation between *oriC*/cell and cell size (µm) was very strong, both in PLF (r = 0.95, *p* < 0.01) and blood (r = 0.98, *p* < 0.05) bacterial populations (Fig. 4c).

In summary, when net population size was increasing, indicating growth, *oriC*/cell and *ori:ter* were high, and cell sizes were large (0–6 hours of infection) (Fig. 4b,c). After 6 hours of infection growth was gradually beginning to slow down, demonstrated by a slight decrease in both *oriC*/cell, *ori:ter* and cell size (µm). The data indicate the presence of a fixed initiation mass, also during infection *in vivo*. Unlike during growth *in vitro*, we did not observe a complete cessation of chromosome replication *in vivo* (*oriC*/cell and *ori:ter* remained >1 at all times during the course of the experiment) (Figs 4b, 5 and 6 and Supplementary Table S1). As demonstrated by the persistent growth heterogeneity (i.e. unbalanced growth), inferring τ from *ori:ter* during growth *in vivo* would be deceptive (Figs 5 and 6). However, *ori:ter*, as demonstrated, may be used as an independent measure of bacterial growth rate during infection *in vivo*.

### Bacterial growth *in vivo*: mouse intravenous (i.v.) septicaemia model

To determine whether the bacterial population found in the bloodstream during peritonitis represented cells growing independently of that at the primary site of infection or not, we attempted to induce primary septicaemia directly via i.v. inoculation of 1 × 10^6^ CFU/ml bacteria (ALO 4783). We were unable to establish an infection, as a complete clearance of bacteria from the bloodstream had occurred prior to the initial sample collection time point; 2 hours post bacterial challenge (Supplementary Fig. S2). No viable bacterial counts were found in blood, PLF or kidneys at any time. However, in the spleen we observed the presence of a stable bacterial population of (mean (SD)) 3.41 (0.03) log_10_ CFU/ml of all animals at all times (2–8 hours post challenge), likely as a result of bacterial bloodstream clearance.

None of the animals presented any signs of distress during the observation period, in contrary to the mouse peritonitis model, where signs of distress were starting to appear from 2 hours of infection.

## Discussion

We have applied and validated two well-established methods for detection of bacterial chromosome replication to shed light on fundamental growth dynamics of *E. coli* during infection *in vivo*. *ori:ter* was determined by qPCR and fluorescence microscopy, respectively, overall with good agreement between methods. The microscopy method has the advantage that it allows for direct observation of *in situ ori:ter* at a single-cell level, which can be used to observe the level of replication heterogeneity within a bacterial population. Also, microscopy can be used to determine cell size, which demonstrated good correlation with the number of replication origins (*oriC*/cell), in agreement with initiation of chromosome replication taking place at a fixed initiation mass under all growth conditions. However, the fluorescence microscopy method requires precedent fluorescent labelling at relevant chromosome loci, along with a certain bacterial quantity for cells to be detected, which is a limitation to the method. The qPCR method, by contrast, reports only the mean *ori:ter* of a population, without regard to any possible heterogeneity. However, this method is less time consuming, uncomplicated and inexpensive, compared to whole genome sequencing. We conclude that both qPCR and fluorescence microscopy are valid methods for measuring *in situ* bacterial growth during infection, and that they complement each other favourably.

Bacterial growth in a closed batch-culture *in vitro* was shown to be directly related to nutrient availability, as anticipated^32,33^. *Ori:ter* levels were high, with a high level of replication heterogeneity, during the first hours of incubation. Subsequently, as the bacterial count increased, we observed a complete cessation of growth within a homogenous population of predominantly non-replicating cells, due to starvation after prolonged propagation^33^. A similar pattern of growth was observed *in vivo* during infection in the mouse peritonitis model. However, unlike during growth *in vitro*, we observed no complete cessation of growth in neither PLF nor blood as the bacterial densities reached their maximum in the terminal stage of infection. At this stage (8 to 10 hours of infection), the stagnant development in bacterial count is deceptive, as it gives the perception of growth cessation. Indeed, a complete cessation of growth might never occur during infection *in vivo*, where bacterial life-sustaining resources presumably are still available as long as the animal is alive. We show in this model that the apparent stationary phase during infection represents fractions of both slowly growing and non-growing cells, conceivably balanced by cells taken out by the host immune system. Hence, it is evident that one cannot extrapolate findings from *in vitro* growth studies to explain pharmacodynamics in relation to bacterial growth during infection *in vivo*, particularly not in regard to the apparent stationary phase. The *in vitro* effect of most commonly used antibiotics, including β-lactams, fluoroquinolones and aminoglycosides, has been shown to correlate to bacterial growth rate^26,27^. Chromosome replication as a readout for bacterial growth rate could form the basis for refined future studies on antibiotic treatment effect as a function of bacterial growth rate during infection *in vivo*.

In the mouse peritonitis model, significantly lower bacterial count densities could be found in the blood, relative to the PLF, yet *ori:ter* and *oriC*/cell remained similar in blood and PLF. These findings, taken together with the observed inability of the same strain to establish an infection upon direct introduction into the bloodstream (as demonstrated by the i.v. septicaemia model), suggest that *E. coli* found outside the primary site of infection (e.g. in the bloodstream) is not constituted by an independently growing population, but rather mirrors the population found at the primary site of infection. This is in consistence with previous *in vivo* studies of *Streptococcus pneumoniae* in the same experimental model, describing the phenomenon as ‘spill-over’^30^. Conceivably, bacterial cells grow at a given rate at the primary site of infection (e.g. the peritoneal cavity), followed by a translocation through lymphatic drainage into the systemic venous system via the thoracic duct and onward to relevant tissues, such as the spleen and kidneys where bacterial phagocytic clearance and filtration, respectively, take place^11,34^. Our findings are in accordance with previous studies indicating that *E. coli*, unlike bacteria such as *Haemophilus influenzae*, do not grow independently intravascularly^8,35,36^. Our data suggest that bacterial cells surviving in the bloodstream remain within the same state of growth as that of bacterial cells at the primary site of infection, without acceleration or significant deceleration; likely as the net result of constant influx- and kill-rate of bacteria. These findings underscore the importance of source control in septicaemia, i.e. locating and ensuring antibiotic availability at the primary site of infection, as this is where active bacterial growth is taking place.

In conclusion, we present data supporting the applicability of both qPCR and fluorescence microscopy as valid methods for determination of bacterial growth rate *in vivo*. The methods could be extrapolated to other infection models and other pathogenic bacteria. Ultimately, it would be evident to pursue the inexpensive and easily accessible qPCR method in a clinical setting to examine bacterial growth rates in infected body fluids, which could prove helpful in evaluating future antibacterial strategies.

## Methods

### Bacterial strains and inoculum preparations

*Escherichia coli* ATCC® 25922™, a clinical isolate from the American Type Culture Collection (Manassas (VA), USA) and CLSI and EUCAST control strain for antibiotic susceptibility testing, was used throughout the study. This strain was applied *in vitro* both as a wild-type and as a genetically modified version expressing fluorescent fusion-proteins at chromosomal sites corresponding to *oriC* and *terC*, respectively (ALO 4783). Only ALO 4783 was applied in the *in vivo* models.

For *in vitro* experiments, inoculum from frozen stock cultures were grown overnight in Lysogeny Broth (LB) broth, shaking 140 rpm, at 37 °C in ambient air. Bacterial counts were quantified by spotting 10 µl of 10-fold serial dilutions made in sterile physiological saline in duplicate on LB agar plates.

For *in vivo* experiments, bacteria were grown from frozen stock cultures overnight at 35 **°**C in ambient air on 5% blood agar plates (SSI Diagnostica, Copenhagen, Denmark), after which inocula were suspended in sterile physiological saline and quantified by measurement of optical density at 546 nm (OD_546_). Porcine mucin (M-2378, Sigma-Aldrich, Munich, Germany) was added as adjuvant to a final concentration of 5% (wt/vol). The mucin stock solution was prepared in physiological saline, sterilised and adjusted to physiological pH before application. The final inoculum (10^6^ CFU/ml) was quantified by spotting 20 µl of serial dilutions in duplicate on selective bromothymol lactose blue agar plates (SSI Diagnostica, Copenhagen, Denmark).

### Construction of double-loci fluorescent-labelled strain

For microscopic visualisation of cellular origins and termini, transgenes were inserted into the ATCC 25922 chromosome, by use of the lambda red recombineering technique previously described^37,38^. P1parS::KAN was PCR amplified from plasmid pMS24^38^ and inserted into the chromosome in the *oriC* region by use of lambda red recombineering plasmid pTP223^39^, using primers 5′-CATCATTAAAGTGCTGTACCGTAACTAACAGAAAGGCCTTAAAGCCGAAGCCTTAAACTT-3′ and 5′-GGGTTAATAGCGATTCAGAGTTCAAGGCCTTCTCCCGGAGGGAACTTCAAGATCCCCTTA-3′. Also, pMT1parS::CHL was PCR amplified from plasmid pGBkD3-parSpMT1^38^ and inserted, by the same method, into the chromosome in the *terC* region using primers 5′- ATATAAATTCTATAATTAGATGTATCTTTCCATTTACGGCGTGTAGGCTGGAGCTGCTTC-3′ and 5′- TCGGTGTGAGATGCTTTACGTCTTCCAAGCCCCCTTCCTTCGTCTTACTGTCGGGAATTC-3′.

Furthermore, the pTrc-mCherry-pMTparB-GFP-P1parB cassette was PCR amplified from pJFM4^40^ and inserted into the chromosomal *attTN7* site at the 3′ end of the *glmS* gene, using the protocol for Tn7 transgene insertion as previously described^41^, resulting in strain ALO 4783. Co-expression of GFP-labelled P1-ParB proteins and mCherry-labelled pMT-ParB proteins^29^ in live cells allowed for visualisation of origins as GFP foci (green) and termini as mCherry foci (red), respectively. Correct insertion of all transgenes was confirmed by PCR.

It should be noted that a substantial part of the LacI operator on pRN010^42^, from which the pTrc-mCherry-pMTparB-GFP-P1parB cassette on pJFM4 originated, had unintentionally been lost during plasmid construction. Thus, the use of IPTG (isopropyl β-D-1-thiogalactopyranoside) for induction of the fluorescence fusion proteins proved redundant.

### *In vitro* experiments

Overnight liquid cultures of both wild-type and genetically modified version of ATCC 25922 with a bacterial density of 10^9^ CFU/ml were diluted 1:10,000 into fresh media and grown with shaking 140 rpm, at 37 **°**C.

Growth was observed by repeated measurements of optical density at 600 nm (OD_600_). Samples for quantification of bacterial count, qPCR analysis and fluorescence microscopy were withdrawn at time points 2, 4 and 6 hours of incubation in the exponential growth phase study, and at times 6, 8 and 10 hours of incubation for the stationary growth phase study, respectively. All samples were immediately set on ice after withdrawal. Both studies were performed in duplicate (i.e. including both wild-type ATCC 25922 as control of growth, and the genetically modified version, ALO 4783) in three independent experiments. As both growth curves and *ori:ter* were the same for both versions of the strain, these results were pooled for statistical analyses. For fluorescence microscopy, only ALO 4783 was applied.

### Experimental animal models

#### Mouse peritonitis model

The mouse peritonitis model has been previously described and studied extensively^43,44^. Here, outbred female NMRI mice (BomTac: NMRI; weight 26–30 g; Taconic, Denmark) were applied throughout the study. The animals were kept in Macrolon type III cages in groups of three and allowed free access to feed and water. Experiments were initiated after an acclimatisation period of 5 days.

Inoculation was performed by intraperitoneal (i.p.) injection of 0.5 ml bacterial suspension containing 10^6^ CFU/ml and 5% (wt/vol) mucin. At various time points of infection, the mice were anaesthetized with a subcutaneous (s.c.) injection of pre-mixed Zolazepam/Tiletamin (Zoletil, Virbac, Kolding, Denmark), Xylasin (Xysol Vet., ScanVet, Fredensborg, Denmark) and Butorphanol (Torbugesic Vet inj., Orion Pharma, Copenhagen, Denmark) prior to blood collection from total cardiocentesis, performed with a 30-gauge needle via subxiphoid access. Blood was stored in EDTA covered micro tubes (Sarstedt, Nümbrecht, Germany). After euthanasia by cervical dislocation, a peritoneal lavage was performed by injecting 2.0 ml of sterile physiological saline i.p. After 1 minute of abdominal massage, the peritoneum was opened and peritoneal lavage fluid (PLF) withdrawn with a pipette. Spleen and both kidneys were surgically removed using sterile procedures and placed in Eppendorf tubes.

All specimens were immediately placed in an insulated 4 **°**C cooling box for transportation and kept on ice at 4 **°**C until application in subsequent tests. Standard bacterial quantification and DNA purification were performed within two hours; fluorescence microscopy the subsequent day. To ensure that bacterial cells in the blood and PLF specimens did not undergo any alterations in any of the growth parameters measured (*ori:ter*, *oriC*/cell or cell size) while kept on ice at a non-permissive growth temperature^45^ post harvesting, we performed a confirmatory *in vitro* experiment where chromosome replication analyses (qPCR and fluorescence microscopy) were performed on exponentially growing cells left on ice for up to 24 hours (Supplementary Materials). The results combined confirmed that storage up to 24 hours on ice at non-permissive growth temperatures did not alter these parameters (Supplementary Fig. S3).

The mouse peritonitis model was repeated in a total of 6 independent experiments, where groups of three cohabitant animals were sacrificed at various combinations of time points; 2 (n = 15), 4 (n = 9), 6 (n = 6) 8 (n = 12) or 10 (n = 9) hours of infection.

Data from repeated experiments were pooled for statistical analyses.

#### Mouse intravenous septicaemia model

To examine the possibility of inducing primary bacteraemia without an established infection outside of the bloodstream, we applied a direct intravenous (i.v.) septicaemia mouse model. Mice, housing, acclimatisation and feeding procedures were identical to that of the mouse peritonitis model. However, here, the mice were inoculated via i.v. injection of 0.2 ml bacterial suspension containing 10^6^ CFU/ml, without adjuvant, into the lateral tail vein. Animals were subsequently handled as above mentioned, with the extraction of blood, peritoneal wash fluid, spleen and kidneys, which were all kept at 4 °C after harvesting. The animals were sacrificed in groups of three biological replicates at 2 (n = 3), 4 (n = 3) and 8 (n = 3) hours of infection.

#### Ethics statement

Mice in both experimental models were regularly observed and scored for signs of distress. Humane end points were constituted by signs of irreversible sickness. The mice would be euthanized upon presentation of any of these signs. All animal experiments were approved by the Danish Animal Experimentation Inspectorate (Licence No. 2014-15-0201-00171) and performed according to institutional guidelines.

### Quantification of bacterial growth

Samples from *in vitro* experiments were spotted (10 µl) in duplicate of serial dilutions on LB agar plates, and the bacterial count was recorded as the mean of two plates after overnight incubation at 37 **°**C in ambient air. The detection limit was 100 CFU/ml.

To the tubes containing spleen or kidneys from the *in vivo* experiments, sterile physiological saline was added to a total volume of 1 ml, after which they were homogenised for 2 minutes at 30 oscillations/sec using a TissueLyser II (Qiagen, US), before being spotted (20 µl) in serial dilutions in duplicate on bromothymol lactose blue agar plates. The required addition of saline before homogenisation of the tissues entailed a dilution factor of approximately 1:1. Blood and peritoneal wash fluid from *in vivo* experiments were spotted directly, without further dilution. Colony counts were performed after overnight incubation at 35 **°**C in ambient air and recorded as the mean of two plates. The detection limit for blood specimens was 50 CFU/ml. The other materials were recorded as CFU/ml of solution.

### Quantitative PCR (qPCR)

*ori:ter*_qPCR_ was calculated as the population mean level of qPCR amplified *oriC* to *terC*, respectively.

Samples from *in vitro* experiments were prepared by pelleting 1 ml of culture by 5 min centrifugation at 15,0000 × g, after which bacterial cells were re-suspended in 100 µl 10 mM Tris pH 7.4 and 900 µl 77% Ethanol and kept at 4 **°**C until qPCR was performed. Here, cells were spun down and re-suspended in serial dilutions of sterile DNA/RNA free water prior to analysis.

Bacterial DNA from blood and peritoneal lavage fluid from *in vivo* experiments were purified for qPCR using QIAamp DNA Mini Kit (51304, Qiagen, Hilden, Germany), according to the manufacturer’s instructions. We were unable to purify adequate quantities of bacterial DNA from spleen and kidney tissue for use in this analysis, possibly due to co-purification of murine DNA and/or low bacterial counts in the tissues.

qPCR was performed as previously described, using primers 5′-CGCAACAGCATGGCGATAAC-3′ and 5′-TTCGATCACCCCTGCGTACA-3′ for partial amplification of the highly conserved *gidA* gene located immediately leftwards of *oriC* (representing the *oriC* region), and primers 5′-TCAACGTGCGAGCGATGAAT-3′ and 5′-TTGAGCTGCGCTTCATCGAG-3′ for partial amplification of the *dcp* gene, located in close proximity to *terC*, opposite the *oriC* region (representing the *terC* region), respectively^28^.

The analysis was performed using Takara SYBR Premix Ex Taq II (RR820A, Takara Bio, Saint-Germain-en-Laye, France) in a BioRAD CFX96 (95 **°**C 30 s, 39 × (95 **°**C 5 s + 58 **°**C 30 s), 95 **°**C 15 s, 60 **°**C 60 s, 95 **°**C −15 s, 65 **°**C −5 s, 95 **°**C − ∞), as previously reported^40^. The *ori:ter*_qPCR_ was calculated using comparative cycle threshold (Ct) analysis; the 2^∆∆Ct^ method^46^. A fixed sample of the same strain grown into late stationary phase, where the population would be expected to have an *ori:ter* corresponding to 1, was used for normalisation in every cycling run.

Each biological replicate was run as a minimum as three technical replicates in each cycling run, and the mean Ct value of the technical triplicates (or more) was used to calculate the *ori:ter*_qPCR_. DNA/RNA-free water was used as negative control template in each run. Furthermore, QIAamp PCR purified blood and peritoneal lavage fluid specimens without viable bacterial growth were regarded as negative controls for blood and peritoneal lavage fluid derived specimens. There was a qPCR detection limit corresponding to approximately 10^3^ CFU/ml for purified blood specimens. Hence, not all purified blood specimens yielded adequate qPCR results. No detection limit was observed in samples from *in vitro* experiments.

### Fluorescence microscopy

*ori:ter*_mic_ was calculated from automated photomicrographic detection of pooled single bacterial cells carrying intracellular fluorescent markers corresponding to *oriC* and *terC*, respectively.

For phase contrast and fluorescence microscopy, bacterial cells were spun down for 2 minutes at 6,000 × g, re-suspended in a small volume of AB minimal medium^47^ and mounted on microscope slides covered with a thin 2% (wt/vol) AB minimal medium agarose pad.

Blood specimens from *in vivo* studies underwent murine cell lysis prior to pelleting, by the addition of cold, sterile water mixed with the sample at a ratio of approximately 1:1 for 1 minute. Harvested tissues were not observed in the microscope.

A total of 500 live bacterial cells were pooled and analysed from each time point, with the exception of certain specimens, where this was not possible due to the quantity of live cells available for microscopy detected being too low (Supplementary Table S1). The *ori:ter*_mic_ was deduced from the mean of *oriC* foci detected divided by the mean of *terC* foci detected.

It should be noted that fractions of the bacterial cells were recorded with a number *oriC* copies ≠ 2^n^ (n = 1, 2, 3). The risk of underestimation of co-localising *oriCs* is a known microscopy resolution limitation in photomicrographic foci quantification^48^. It is unlikely that the bacterial cells propagating *in vitro* underwent asynchronous replication initiation^4^. Accordingly, cells reported with an *oriC*/cell of 3 in all likelihood represent 4 *oriC*/cell, and cells reported with an *oriC*/cell > 4 in all likelihood represent 8 *oriC*/cell (Fig. 3). Since less is known about chromosome replication during infection *in vivo*, we cannot rule out any possible replication initiation asynchrony in bacterial populations from the *in vivo* experiments (Figs 5 and 6).

Medial axis cell length (µm) was recorded by automated detection and used as surrogate measure for cell mass.

Images were acquired with a Zyla 5.5 sCMOS camera attached to a Nikon Eclipse Ti-E inverted microscope and analysed with NIS-ELEMENTS and MicrobeJ software for automated bacterial cell and fluorescent foci detection and quantification^49^.

### Statistical analyses

Bacterial quantification data were log_10_ transformed prior to analysis. D’Agostino and Pearson omnibus normality test was applied to all data sets. In general, the bacterial count and the qPCR data sets represented a normal distribution. Cell length and numbers of *oriC* foci detected by microscopy did not all meet the assumptions for normal distribution. Linear regression analysis was used to compare bacterial counts or *ori:ter* between different materials harvested in the *in vivo* experiments. Additionally, two-way ANOVA followed by Sidak’s multiple comparisons test was applied within the same data sets to test difference between blood and peritoneal *ori:ter* at each time point. Pearson’s correlation coefficient was applied to estimate correlation between cell length (µm) and *oriC*/cell. The Kolmogorov-Smirnov test was applied to test differences in *oriC*/cell distributions between bacterial populations in the blood and PLF, respectively, at different time points. Bland-Altman analysis was performed to evaluate agreement between the qPCR and the microscopy method for detection of *ori:ter*. A two-tailed *p* value < 0.05 was considered significant.

GraphPad Prism version 7 (GraphPad Software, CA, USA) was applied for statistical analysis and illustration.

## Electronic supplementary material

Supplementary information

## Data Availability

The datasets generated during and/or analysed during the current study are available from the corresponding author on reasonable request.
