# Supplementary material for: Chromosome replication as a measure of bacterial growth rate during Escherichia coli infection in the mouse peritonitis model
Source: Sci Rep. 2018 Oct 8;8:14961. doi: 10.1038/s41598-018-33264-7 (PMC6175860; doi:10.1038/s41598-018-33264-7)
Supplement: Supplementary file 1 — Supplementary information [file 41598_2018_33264_MOESM1_ESM.pdf]

Supplementary Information

Chromosome replication as a measure of bacterial growth rate during *Escherichia coli* infection in the mouse peritonitis model

Authors: Maria Schei Haugan[1, 2], Godefroid Charbon[1], Niels Frimodt-Møller[2, 3], Anders Løbner-Olesen[1, 3 *]

Affiliations: [1] Department of Biology, University of Copenhagen, Copenhagen, Denmark; [2] Department of Clinical Microbiology, Rigshospitalet, Copenhagen, Denmark

[3] Co-senior authors

*Corresponding author

Supplementary Methods

***In vitro* experiment**

To ensure that bacterial cells in the biological specimens (blood and peritoneal lavage fluid (PLF)) harvested in the *in vivo* experiments and subsequently applied in chromosome replication analyses (qPCR and fluorescence microscopy) did not undergo any alterations in growth post harvesting, while kept on ice at 4°C, we performed an additional *in vitro* experiment where chromosome replication analyses were performed on exponentially growing cells after various amounts of time on ice.

An overnight liquid culture of ALO 4783 with a bacterial density of 10^9 CFU/ml was diluted 1:10,000 into fresh Lysogeny Broth (LB) and grown with shaking 140 rpm, at 37°C. Growth was observed by repeated measurements of optical density at 600 nm (OD_{600}). At OD_{600} of approximately 0.4, samples were withdrawn in triplicates and placed on ice at 4°C. To mimic the change in growth environment that the bacterial cells in the peritoneum underwent (sterile isotonic saline suspension; see Methods in article), half of the samples were spun down for 3 minutes at 6000 rpm, after which the pellet was re-suspended in sterile isotonic saline (NaCl), before being placed on ice. The other half was placed on ice without change of media (LB). At t = 20 min, 1 h, 2 h and 24 h the triplicate samples withdrawn from ice and analysed by qPCR ($ori{:}ter_{qPCR}$) and fluorescence microscopy (*oriC*/cell and cell length (µm)), as described in Methods in the article. Reference samples (t = 0 h) were analysed immediately, without standing on ice.

Kruskal-Wallis test followed by Uncorrected Dunn's test was performed on the data sets to test any difference compared to the reference samples. A two-tailed p value < 0.05 was considered significant.

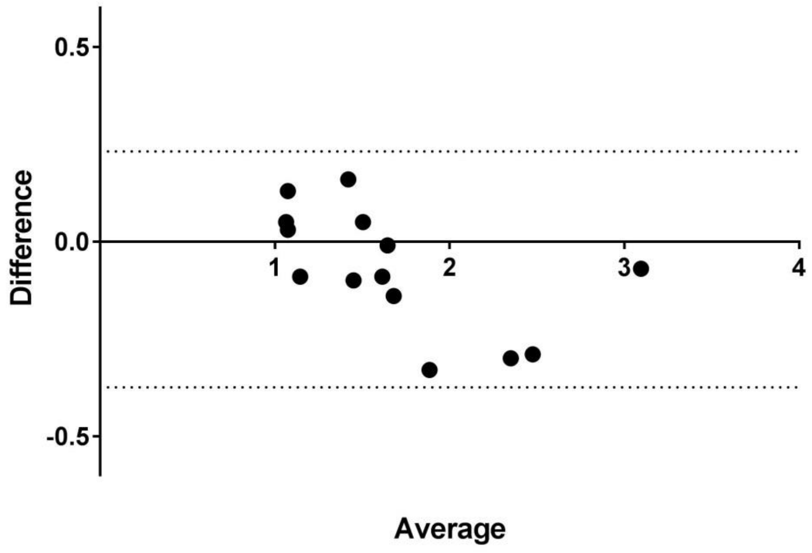

Supplementary Figure S1. Bland-Altman plot of agreement between the two methods for detection of replication ratio (qPCR and fluorescence microscopy). The plot is presented as difference (*ori:ter*$_{mic}$ - *ori:ter*$_{qPCR}$) versus average (*ori:ter*$_{mic}$ and *ori:ter*$_{qPCR}$) for *in vitro* and *in vivo* (mouse peritonitis model) data combined. There was a bias (SD) of 0.07 (0.15) and 95% limits of agreement (presented as dotted lines) from -0.37 to 0.23. There was no systemic variation over the range of measurements. Data from 2 (blood) and 4 (blood and peritoneal lavage fluid (PLF)) hours of infection (*in vivo)* were not included in the analysis due to insufficient number of microscopically detected cells (n < 100, see Supplementary Table S1).

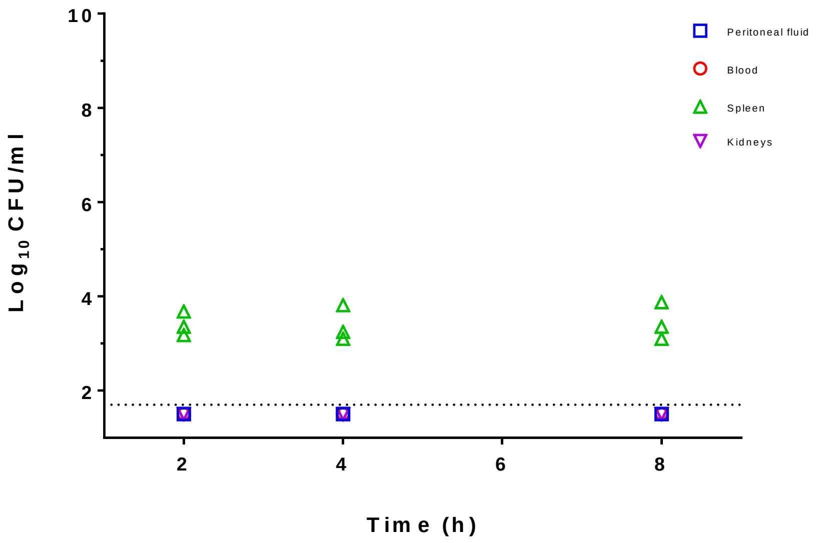

Supplementary Figure S2. Bacterial growth (ALO 4783) at various anatomical sites after attempted induction of septicaemia in the mouse intravenous (i.v.) septicaemia model (*in vivo*). Each symbol represents the mean bacterial count ($\log_{10}$ CFU/ml) per specimen (animal), withdrawn at 2 (n = 3), 4 (n = 3) and 8 (n = 3) hours (h) post inoculation, respectively. Dotted line represents the limit of detection.

(a)

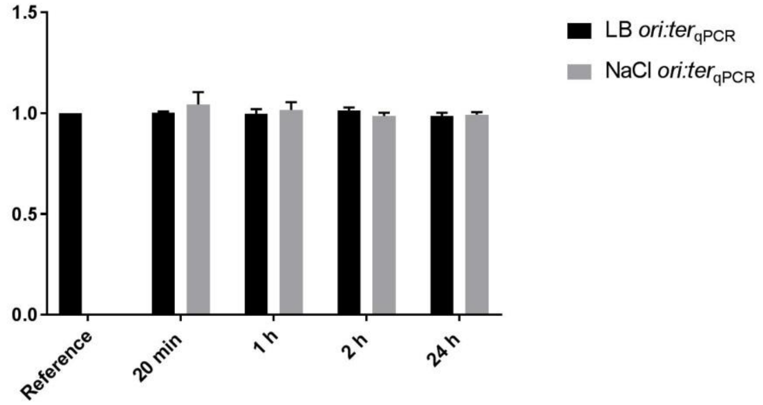

(b)

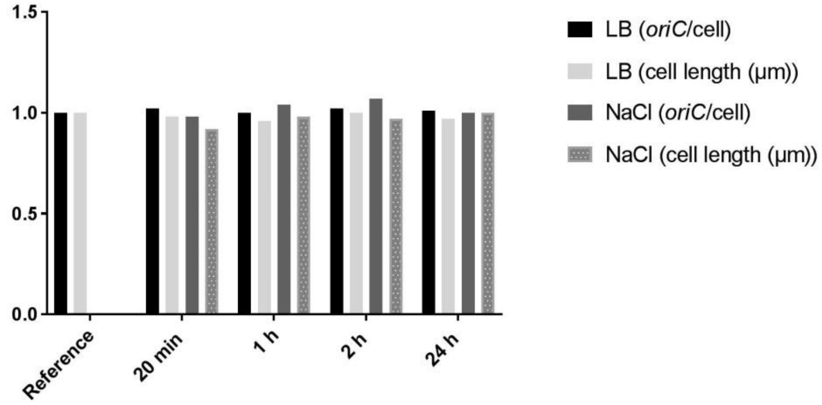

Supplementary Figure S3. Bacterial chromosome replication status of bacterial cells (ALO 4783) withdrawn during exponential growth in LB batch culture and left standing on ice at 4°C. **(a)** *ori:ter*$_{qPCR}$ of triplicate samples left on ice (20 min, 1h, 2h or 24h), with (LB) or without (NaCl) change of media. Data are presented as mean (SD), relative to the reference (0h). n = 3 per time point. There was no significant difference ($P > 0.05$) in absolute *ori:ter*$_{qPCR}$ between any of the samples left on ice and the reference samples. **(b)** Microscopically detected bacterial cell length (µm) and *oriC*/cell from triplicate samples left on ice (20 min, 1h, 2h or 24h), with (NaCl) or without (LB) change of media. Data are presented as the mean of a total of 100 pooled microscopically detected cells, relative to the reference (0h). There was no significant difference ($P > 0.05$) in absolute *oriC*/cell or cell length (µm) between any of the samples left on ice and the reference samples, with the exception of a marginally significant difference ($P < 0.05$) in cell length(µm) between the NaCl samples left on ice for 20 minutes and the reference samples. As the same bacterial cells (NaCl 20 min) do not differ from the controls in neither *oriC*/cell nor *ori:ter*$_{qPCR}$, the latter finding is not considered relevant.

Supplementary Table S1 I Overview of fluorescence microscopy, qPCR and bacterial count results from *in vitro* and *in vivo* (mouse peritonitis model) experiments

Sample origin and time point (hours of incubation / infection)	Fluorescence microscopy					qPCR		Bacterial counts	
	***oriC*/cell** Mean (SD)	***terC*/cell** Mean (SD)	***ori:ter*$_{mic}$**	**Cell length (µm) Mean (SD)**	**n[a]**	***ori:ter*$_{qPCR}$** Mean (SD)	**n[b]**	**Log_{10} CFU/ml** Mean (SD)	**n[c]**
Inoculum used for *In vitro* experiments	1.23 (0.44)	1.11 (0.33)	1.10	2.57 (0.57)	500	1.04 (0.07)	6	9.31 (0.36)	6
In vitro, 2h	3.05 (1.22)	1.31 (0.46)	2.33	4.03 (1.05)	147	2.62 (0.41)	6	5.61 (0.10)	6
In vitro, 4h	3.48 (1.13)	1.14 (0.35)	3.06	4.23 (1.02)	500	3.13 (0.55)	6	7.85 (0.23)	6
In vitro, 6h	1.42 (0.60)	1.01 (0.09)	1.40	3.45 (0.78)	500	1.50 (0.19)	12	9.22 (0.23)	12
In vitro, 8h	1.17 (0.39)	1.02 (0.15)	1.14	2.59 (0.68)	500	1.01 (0.07)	6	9.54 (0.08)	6
In vitro, 10h	1.14 (0.35)	1.03 (0.18)	1.09	2.24 (0.51)	500	1.06 (0.09)	6	9.61 (0.14)	6
Inoculum used for *in vivo* experiments	1.15 (0.37)	1.05 (0.22)	1.09	2.43 (0.57)	500	1.19 (0.09)	6	6.05 (0.09)	6
In vivo, 2h, Peritoneal lavage fluid (PLF)	2.46 (1.31)	1.12 (0.33)	2.20	3.96 (1.16)	133	2.50 (0.36)	15	5.88 (0.31)	15
In vivo, 2h, Blood	3.00 (0.89)[d]	ND[d/e]	ND[d/e]	4.06 (1.06)[d]	9[d]	2.41 (0.45)	12	4.64 (0.90)	15
In vivo, 4h, PLF	2.16 (0.97)[d]	1.16 (0.37)[d]	1.85[d]	3.68 (1.21)[d]	55[d]	1.93 (0.28)	9	6.30 (0.66)	9
In vivo, 4h, Blood	2.82 (1.33)[d]	1.72 (0.65)[d]	1.63[d]	3.97 (0.53)[d]	11[d]	1.58 (0.32)	8	4.87 (0.77)	9
In vivo, 6h, PLF	2.15 (1.03)	1.25 (0.52)	1.72	3.18 (0.92)	132	2.06 (0.12)	6	6.83 (0.58)	6
In vivo, 6h, Blood	2.10 (0.79)	1.31 (0.49)	1.61	3.09 (0.73)	164	1.76 (0.22)	5	5.32 (1.19)	6
In vivo, 8h, PLF	1. 90 (0.78)	1.16 (0.39)	1.64	3.14 (0.77)	500	1.66 (0.30)	12	7.79 (0.81)	12
In vivo, 8h, Blood	1.99 (0.82)	1.27 (0.48)	1.57	3.17 (0.72)	157	1.67 (0.29)	12	6.83 (1.10)	12
In vivo, 10h, PLF	1.64 (0.76)	1.06 (0.28)	1. 53	2.76 (0.76)	500	1.48 (0.17)	9	7.54 (1.07)	9
In vivo, 10h, Blood	1.64 (0.75)	1.09 (0.33)	1.51	2.87 (1.04)	500	1.34 (0.24)	9	7.09 (1.88)	9

[a] number of pooled microscopically detected bacterial cells

[b] number of biological replicates yielding reproducible qPCR results

[c] number of biological replicates analysed for qPCR and bacterial count

[d] data are subject to uncertainty due to low number (n < 100) of microscopically detected cells

[e] ND: not determined, due to insufficient fluorescent (mCherry) signal
